# Acute respiratory distress syndrome caused by varicella pneumonia in immunocompetent adult: Clinical case

**DOI:** 10.1016/j.amsu.2021.01.080

**Published:** 2021-01-26

**Authors:** Mohammed Aabdi, Mimouni Hamza, Lezreg Moussa, Bkiyar Houssam, Housni Brahim

**Affiliations:** Intensive Care Unit, Faculty of Medicine and Pharmacy of Oujda, Mohammed VI University Hospital, Mohammed I University, Oujda, Morocco

**Keywords:** Varicella, Pneumonia, Acute respiratory distress syndrome, Immunocompetent, Case report

## Abstract

**Introduction:**

Varicella zona infection is a rare condition in immunocompetent adults. It can lead to severe and lethal complications including Varicella pneumonia that can rapidly progress to acute respiratory distress syndrome a rare and life-threatening situation.

**Clinical case:**

A 63 years old man was admitted to the intensive care unit for pneumonia with generalized papulovesicular lesions. After investigations, the diagnosis of Varicella pneumonia complicated with acute respiratory distress syndrome was maintained and the patient was put on mechanical ventilation, and despite proper management (antiviral treatment; protective ventilation and prone position) the patient died 48 hours after his admission.

**Conclusion:**

Despite its rarity, Varicella pneumonia can be a life-threatening situation in immunocompetent adults. The diagnosis must be evoked when the patient presented with respiratory manifestations with dermatologic lesions.

## Highlights

• Varicella is a highly contagious disease caused by the initial infection with varicella-zoster virus (VZV).
• The clinical symptomatology of Varicella infection is usually mild, however, it can manifest with serious complications including varicella pneumonia in immunocompromised patients.
• In this paper we describe a rare clinical case of pnumonia varicella complicated with acute respiratory distress syndrom in immunocompetent adult.

## 1 Introduction

Varicella is a highly contagious disease caused by Varicella-Zoster Virus, it's a common childhood self-limited disease but it can be a life-threatening situation in immunocompromised patients [[1], [2], [3]]. And despite its rarity, Varicella zona infection can lead to severe lethal complications in immunocompetent persons including varicella pneumonia that can rapidly progress to an acute respiratory distress syndrome despite conventional support [[4], [5], [6]].

In this paper; we will report the clinical case of a 63 years old man, with no medical history, admitted to the critical care unit for acute respiratory distress syndrome due to Varicella pneumonia. We will discuss the respiratory manifestations of Varicella-Zoster virus, the diagnosis and the treatment.

## 2 Clinical case

A 63-year-old man with a history of smoking 23 packets/years weaned for 10 years, with no other medical history was admitted to the emergency room for dyspnea, 5 days after a diffuse rash that started in his scalp.

**Fig. 1:**
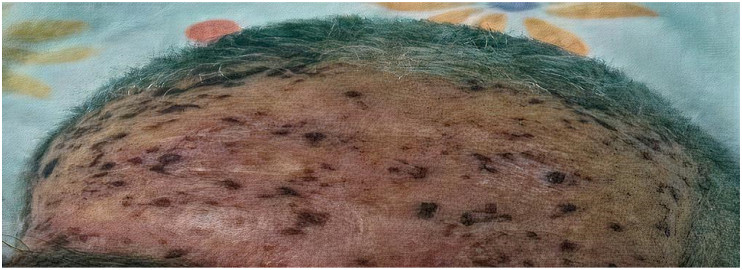
Image of the patient showing generalized papulo-vesicular lesions with certain lesions with a necrotic center.

The physical examination on his admission was as follow: fever at 38.5 °C, polypnea at 30 cycles/minute, pulse oxymetry at 65% under high concentration mask at 15 L/min, bilateral rhonchi, perioral and extremists cyanosis with signs of respiratory exhaustion: paradoxical respiration, high blood pressure at 180/110 mmHg, heart rate at 110 beats/min and generalized papulovesicular lesions with certain lesions with a necrotic center (Fig. 1).

High flow nasal cannula treatment was initiated with the flow of 80l/min and the inspiratory fraction of oxygen at 100% with no improvement, the patient was intubated.

The arterial blood gas after intubation was as followed: pH 7.23, PaO2 42 with FiO2 of 100% and PaO2/FiO2 of 42, PaCO2 65, HCO3- 14 and lactates at 4.05.

The complete blood count: hyperleukocytosis at 11,460/μm, lymphopenia at 450/mm3, thrombocytopenia at 110,000 μm, CRP at 113 mg/l, procalcitonin at 0.82, hepatic cytolysis with ASAT and ALAT 64 and 45 higher than limit respectively, the elevation of lactate dehydrogenase (LDH) at 1289 IU/l, hypoalbuminemia at 27 g/l, urinary antigen test for streptococcus pneumoniae and legionella pneumonia were negative, human immunodeficiency virus HIV testing was negative, RT-PCR (reverse transcriptase-polymerase chain reaction) for Covid-19 and H1N1 were negative, and Varicella zona virus serology was positive.

**Fig. 2:**
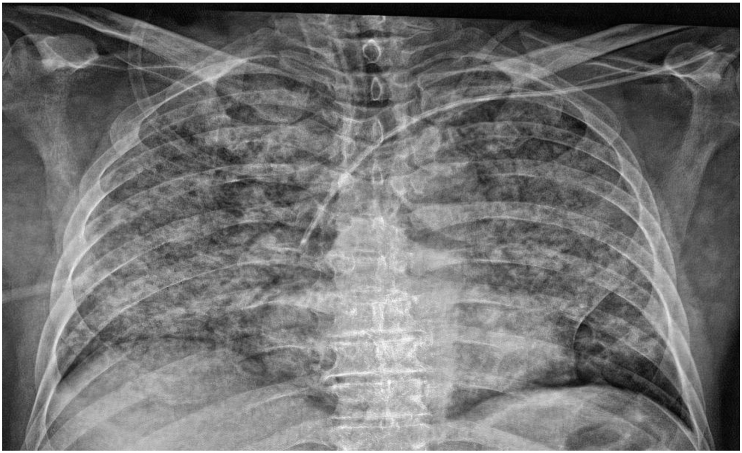
Chest X-ray showed the presence of bilateral and diffuse alveolar interstitial opacities in the two pulmonary fields.

The chest X-ray showed the presence of bilateral and diffuse alveolar interstitial opacities in the two pulmonary fields (Fig. 2).

The diagnosis of acute respiratory distress syndrome caused by Varicella pneumonia was maintained by association of respiratory manifestations, dermatological lesions and positive serology for Varicella Zona Virus.

Initial treatment with acyclovir (10 mg/kg every 8 h) was initiated.

The evolution was fatal, despite prone position, curare perfusion and optimal sedation, the patient died after 48 hours of his admission.

## 3 Discussion

Varicella is a highly contagious disease caused by the Varicella-Zoster Virus with a worldwide distribution, it is a member of the « *Herpesviridae* » family of DNA virus which can cause lytic and latent infections [1,7,8]. It is acquired by inhalation of infected saliva droplets or rarely a direct contact with skin lesions [7].

It occurs in all countries with mild self-limiting symptoms lasting a few days and providing lifetime immunity but it can cause serious complications among high-risk populations with lethality of 7000 deaths/year [7,9].

Clinical signs of varicella are usually mild including intense prurit, blasters on palms and genitals; small painful and itchy ulcers on the oral cavity appearing 21 days after exposure to the virus [7].

The varicella reinfection is rare, causing serious painful illness in immunocompromised individuals with high a mortality rate [7,10,11].

Varicella pneumonia is the most common complication of adult Varicella with an incidence of 1/400 with risk factors such as smoking, immunosuppression and preexistent lung disease with a mortality rate of 10%–30% overall and up to 50% in those on mechanical ventilation [12,13].

Respiratory symptoms of Varicella pneumonia began 1–7 days after installation of the rash, they are usually mild with few respiratory symptoms such as dry cough, hemoptysis, thoracic pain, dyspnea, fever, and even acute respiratory distress [14,15].

Varicella induced acute respiratory distress syndrome is very rare but a life-threatening situation [16,17].

Radiologic images find ill-defined confluent nodules in 2 pulmonary fields, ground-glass opacities surrounding the nodules or diffuse, hilar lymphadenopathy, and pleural fluid [18,19].

Diagnosis is based on clinical findings, but laboratory tests are still essential in cases of atypical cases or disseminated infection, the virus is searched by molecular tests on skin lesion fluid, blood and respiratory samples and serologic diagnosis is useful to identify unprotected individuals and distinguish primary infection from reactivation [20].

Treatment of pulmonary chickenpox consists of antiviral drugs; acyclovir 10mg/kg/8 hours for a period of 7–10 days [7].

Purified immunoglobulins with anti-VZV antibodies can be administered intramuscularly 96 hours to 10 days after rash apparition [20].

## 4 Conclusion

Despite its rarity, varicella pneumonia can be a life-threatening situation in immunocompetent adults, with the possibility to develop an acute respiratory distress syndrome.

The diagnosis should be evoked in patients with respiratory distress with dermatologic lesions to start early management and avoid serious complications including death.

The work has been reported in line with the CARE 2018 criteria [21].

## Ethical approval

The paper reflects the authors' own research and analysis in a truthful a complete manner.

## Funding

This research did not receive any specific grant from funding agencies in the public, commercial, or not-for-profit sectors.

## CRediT authorship contribution statement

• Dr. AABDI MOHAMMED: Writing - original draft, Conceptualization, Methodology, Software, Validation, Formal analysis, Visualization.
• DR. MIMOUNI HAMZA: Resources, Data curation, Software. final approval of the version to be submitted
• Dr. LAZREG MOUSSA: Writing - review & editing, Formal analysis, Visualization. final approval of the version to be submitted
• Pr. BKIYAR HOUSSAM: Writing - review & editing, Visualization. final approval of the version to be submitted
• Pr. HOUSNI: Project administration, Visualization, Writing - review & editing, Resources, Conceptualization, Methodology, Validation. final approval of the version to be submitted

## Consent

We have obtained the consent from the patient's wife for publication.

## Guarantor

AABDI Mohammed MIMOUNI Hamza

## Declaration of competing interest

The authors certify that they have NO affiliations with or involvement in any organization or entity with any financial interest (such as honoria; educational grants; participation in speakers’ bureaus; membership, employment, consultancies, stock ownership, or other equity interest; and expert testimony or patent-licensing arrangements). Or non-financial interest (such as personal or professional relationships, affiliations, knowledge or beliefs) in the subject matter or materials discussed in this manuscript.
